# Structure-Activity and Lipophilicity Relationships of Selected Antibacterial Natural Flavones and Flavanones of Chilean Flora

**DOI:** 10.3390/molecules22040608

**Published:** 2017-04-10

**Authors:** Javier Echeverría, Julia Opazo, Leonora Mendoza, Alejandro Urzúa, Marcela Wilkens

**Affiliations:** Facultad de Química y Biología, Universidad de Santiago de Chile, Santiago 9170022, Chile; juliaopazo@yahoo.com (J.O.); leonora.mendoza@usach.cl (L.M.)

**Keywords:** antibacterial flavonoids, structure-activity relationship, lipophilicity

## Abstract

In this study, we tested eight naturally-occurring flavonoids—three flavanones and five flavones—for their possible antibacterial properties against four Gram-positive and four Gram-negative bacteria. Flavonoids are known for their antimicrobial properties, and due their structural diversity; these plant-derived compounds are a good model to study potential novel antibacterial mechanisms. The lipophilicity and the interaction of antibacterial compounds with the cell membrane define the success or failure to access its target. Therefore, through the determination of partition coefficients in a non-polar/aqueous phase, lipophilicity estimation and the quantification of the antibacterial activity of different flavonoids, flavanones, and flavones, a relationship between these parameters was assessed. Active flavonoids presented diffusion coefficients between 9.4 × 10^−10^ and 12.3 × 10^−10^ m^2^/s and lipophilicity range between 2.0 to 3.3. Active flavonoids against Gram-negative bacteria showed a narrower range of lipophilicity values, compared to active flavonoids against Gram-positive bacteria, which showed a wide range of lipophilicity and cell lysis. Galangin was the most active flavonoid, whose structural features are the presence of two hydroxyl groups located strategically on ring A and the absence of polar groups on ring B. Methylation of one hydroxyl group decreases the activity in 3-*O*-methylgalangin, and methylation of both hydroxyl groups caused inactivation, as shown for 3,7-*O*-dimethylgalangin. In conclusion, the amphipathic features of flavonoids play a crucial role in the antibacterial activity. In these compounds, hydrophilic and hydrophobic moieties must be present and could be predicted by lipophilicity analysis.

## 1. Introduction

Among natural products, flavonoids are widely distributed in plants, showing a broad range of biological activities [1], like antioxidant [2,3,4,5], anticarcinogenic [6,7], and antimicrobial [8,9,10,11,12] effects. The basic flavonoid structure is represented by a C6-C3-C6 carbonated skeleton with different bonds between the phenylpropanoid and acetate units, being classified as isoflavonoids, neoflavonoids, and flavonoids [9]. They can also be hydroxylated, glycosylated, sulfated, and methylated, resulting in a great variety of different compounds [9,13,14]. Based on this structure diversity, and their activity against Gram-positive and Gram-negative bacteria [9], this family of compounds constitutes an especially interesting group for the development of new antibacterial drugs.

An antibacterial compound requires a special set of physicochemical properties to access the site of action inside the bacterial cell [15]. The cell wall represents a complex barrier to the entrance of compounds. Gram-positive bacteria possess a thick peptidoglycan layer and, in the case of Gram-negative bacteria, in addition to the inner membrane and the peptidoglycan layer, an outer membrane represents an additional barrier for many small molecules. A compound whose site of action is inside the cell must interact with the cell membrane to gain access to its target in the required critical concentration, and a lipophilic moiety is important for this interaction [16,17]. The permeability of small molecules is correlated with their solubility in nonpolar solvents and their solubility in water [18]. To cross the bacterial membrane, the small molecule is first solvated in the extracellular media, immersed in the alkylated part of the lipid bilayer, finally diffusing to the cytoplasmic side, where it is again solvated. This behavior can be related experimentally with the partition coefficient of the compound in a non-polar/aqueous phase, and theoretically with the lipophilicity. Using this latter approach in filifolinol derivatives, good qualitative correlation was shown between lipophilicity and antimicrobial activity [19], the relationship between the lipophilicity of flavonoid phytoalexins analogues and the growth inhibition of two fungi (*Aphanomyces euteches* and *Fusarium solani*), and with the minimal inhibitory concentration against the Gram-positive bacteria *Streptococcus faecium* [16]. In the last cases, they found that the flavonoid activity was different depending on the presence of one hydroxyl group, and that at least some lipophilicity was needed for both the antifungal and antibacterial activities. Retrochalcones were more active against bacteria when the hydrophobicity was improved by the addition of prenyl groups on ring B [20]. Structurally, the position and number of hydroxyl groups on the flavonoid rings were also important for the antioxidant activity [2,21].

In the design of novel drugs, a good understanding of the properties that are responsible for their activity is an important goal in their development. A second important aspect for the potential antibacterial activity of flavonoids is their permeation through the cell walls of microorganisms [15] and their interaction with the cell membrane [22]. This property is associated with their solubility in the lipid bilayers that protect bacterial cells, and may be evaluated by the assessment of the drug’s lipophilicity [17] by means of a fluorescence polarization method, analyzing the interaction of flavonoids with liposomal membranes, showing that the antibacterial activity of flavonoids against *Escherichia coli* depends on the molecular hydrophobicity and the charges on C-3 atom.

The requirement of a hydrophobic behavior of these molecules is in contrast with their need of having hydrophilic phenolic groups for their activity. A proper method of evaluating these compounds should incorporate these opposing features in the measurement of some physico-chemical properties that might correlate with their activity. The determination of their diffusion coefficients in a protic solvent takes into account these opposing factors. Their diffusion in a hydroxylic solvent should depend not only on their size, but also on their intermolecular interactions with the solvent. The presence of hydrogen-bond-forming groups in the molecule increases these interactions, whereas hydrophobic groups tend to reduce them.

In previous publications the diffusion coefficients of some flavonoids in methanol were measured by the Taylor-Aris technique [23], which has been used for estimating drug partition in biological systems. The results allowed an analysis of the solute-solvent interactions of these systems to be made, that was supported by dynamics simulations [24]. There is no rigorous data published elsewhere about a correlation between the lipophilicity and antibacterial activity of flavonoids, and how the lipophilicity affects compound-membrane interaction.

In the present communication, we applied the Taylor-Aris technique to measure the diffusion coefficients and the theoretical lipophilicity estimation of flavonoids, specifically flavones and flavanones, searching for correlations between these physico-chemical parameters and their activity as antimicrobial agents against Gram-negative and Gram-positive bacteria.

## 2. Results

### 2.1. Antibacterial Activity Determination

**Figure 1:**
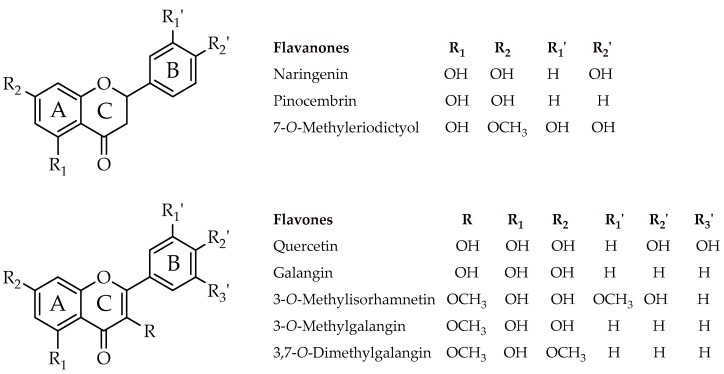
Flavonoids used in this study.

Although flavonoids are known to possess antibacterial activity, the effect of oxygenation patterns on the potency has not been systematically investigated. In the present study, three flavanones and five flavones (see Figure 1 for structures) were tested against a panel of microorganisms, including four Gram-negative bacteria (*Enterobacter cloacae*, *Escherichia coli*, *Klebsiella pneumoniae*, and *Proteus mirabilis*) and four Gram-positive bacteria (*Bacillus cereus*, *B. subtilis*, *B. coagulans*, and *Staphylococcus aureus*). Pure methanol showed no inhibition; for this same reason it was used as a negative control. The results indicated that each compound showed more or less pronounced antibacterial potencies, except 3,7-*O*-dimethylgalangin, affecting both Gram types. However, the latter group of bacteria appeared to be more sensitive for most of the flavonoids tested, particularly flavone, regardless of the oxygenation pattern. Among the flavones, in solid media, galangin and 3-*O*-methylgalangin were the most active compounds against Gram-positive and Gram-negative bacteria whereas, among the flavanones, the most active against representatives of both groups were pinocembrin and 7-*O*-methyleriodictyol. The minimum inhibitory concentration (MIC) for the different flavonoids was determined (Table 1) as the amount of compound in μg in the 5 μL aliquot deposited over the bacterial lawn. Galangin and 3-*O*-methylgalangin showed a MIC that ranged from 0.25 to 1 μg/mL for all tested bacteria. The other flavonoids needed higher amounts to obtain a transparent halo of bacterial growth inhibition, or they were inactive at all concentrations up to 4 μg/mL of applied compound, as shown for 3,7-*O*-dimethylgalangin. Gram-positive bacteria were slightly more sensitive to the flavonoids than Gram-negative bacteria.

**Figure 2:**
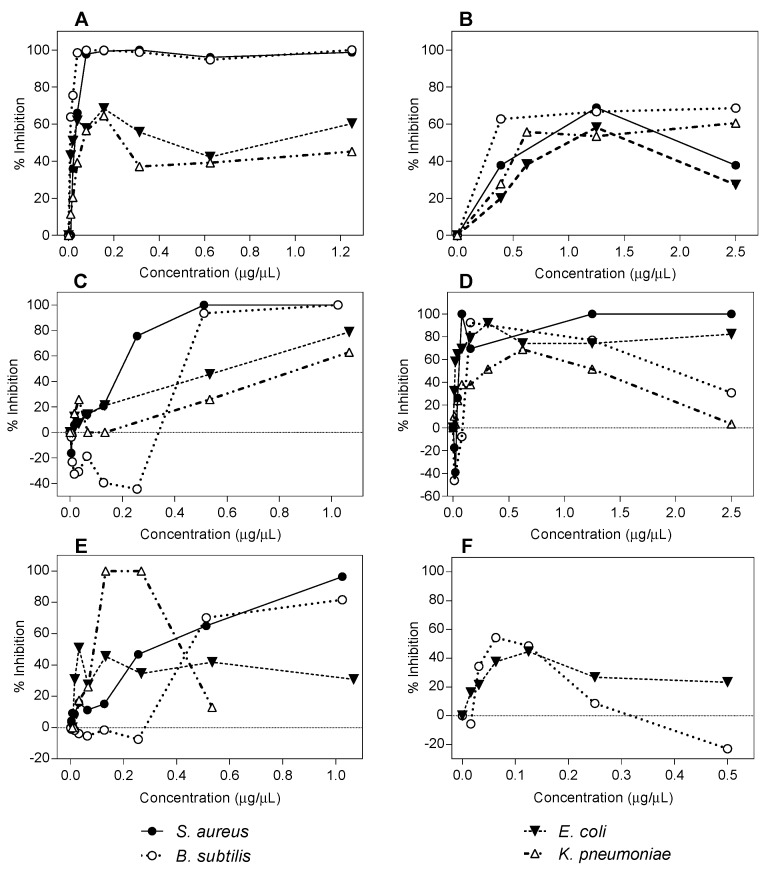
Effect of flavonoids on the stationary phase bacterial growth. Bacteria (2 × 10^8^ ufc/mL, 10 μL in a final volume of 150 μL) were incubated stationary at 37 °C for 18 h in the presence of different concentrations of flavonoids in lysogeny broth (LB) in 96-well micro dilution plates. The final optical density (O.D.) in each well was measured in an Enzyme-Linked ImmunoSorbent Assay (ELISA) detector at 540 nm. (**A**) pinocembrin; (**B**) 7-*O*-methyleriodictyol; (**C**) galangin; (**D**) quercetin; (**E**) 3-*O*-methylgalangin; and(**F**) 3,7-*O*-dimethylgalangin. The graphs are representative of the results of three replicates.

The inhibitory activities of all flavonoids were also tested in liquid media in a 96-well microdilution test at different final concentrations, and only some representative examples are shown in Figure 2. The graphs show the relationship between the maximal growths determined as the optical density reached by each bacteria in the presence of different compound concentrations. Gram-positive bacteria showed a higher susceptibility than Gram-negative bacteria.

Pinocembrin completely inhibited the growth of *S. aureus* and *B. subtilis*, resulting in zero turbidity with a concentration of 0.01 μg/μL (Figure 2A). Higher concentrations were needed with galangin and quercetin (Figure 2C,D, respectively). Gram-negative bacteria *K. pneumoniae* was more sensitive to all compounds than *E. coli* (triangles in Figure 2A–D), and galangin inhibited the cell growth almost completely at higher concentrations (Figure 2C). The presence of 3,7-*O*-dimethylgalangin in the culture media did not alter bacterial growth of *B. subtilis* and *S. aureus*, as shown in Figure 2F for both type of bacteria, and 7-*O*-methyleriodictyol reached 50% growth inhibition at a concentration of 1.25 μg/μL (Figure 2B). For some compounds, higher concentrations exhibited higher optical density values than at low concentrations. It has been shown that flavonoids dissolved in organic solvents precipitate when mixed in polar solutions, diminishing the contact with the bacterial cells [9,25]. Furthermore, it has been determined that some compounds may act as aggregators of bacterial cells altering the turbidity and viability results [9,26,27].

**Figure 3:**
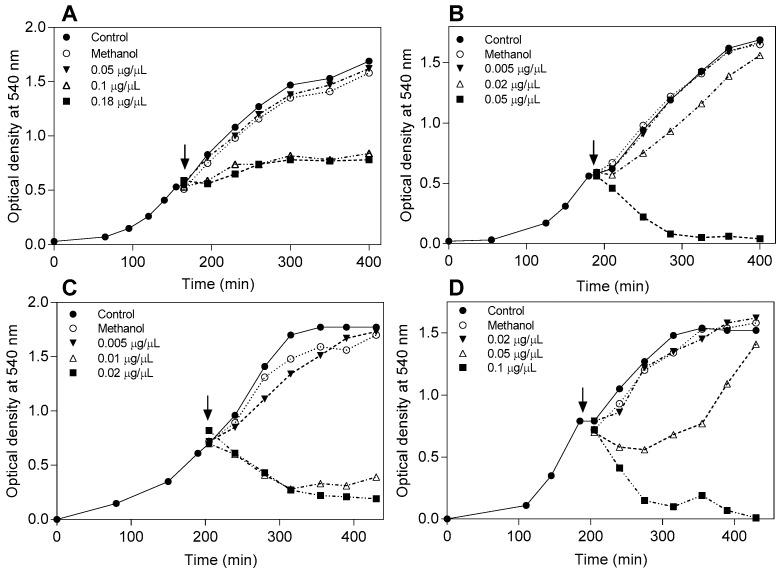
Effect of flavonoids on bacterial exponential growth and viability. 10 mL *E. coli* (**A**) and *B. subtilis* (**B**–**D**) cultures at O.D._540 nm_ 0.5 (shown by the arrow) were treated with different concentrations (indicated in the figure) of pinocembrin (**A**,**B**), 3-*O*-methylgalangin (**C**), and 7-*O*-methyleriodictyol (**D**), or the cells were not treated or they were treated with methanol alone. The graphs are representative of the results of three replicates.

To determine the effect of some flavonoids on the growth curve of *E. coli* and *B. subtilis*, compounds were added to exponentially-growing cultures (Figure 3). Pinocembrin showed a slight inhibitory effect against the Gram-negative bacteria (Figure 3A) at the highest concentration used (0.18 μg/μL). On the other hand, all assayed compounds, pinocembrin, 3-*O*-methylgalangine, and 7-*O*-methyleridictyol, decreased the culture turbidity and cell viability of the Gram-positive bacteria *Bacillus subtilis* (Figure 3B–D), with the lowest concentration for 3-*O*-methylgalangin (0.01 μg/μL) and the highest for 7-*O*-methyleridictyol (0.1 μg/μL). Compounds, like 7-*O*-methyleridictyol and 3-*O*-methylsorhamnetin, needed 0.1 and 0.18 μg/μL, respectively, to cause the same effect on optical density with *B. subtilis* (data not shown). However, 3,7-*O*-dimethylgalangin did not affect the bacterial growth (data not shown). In Figure 3, for some compounds, bacterial growth was affected and, afterwards, it was reestablished. This behavior could be explained by the stress response of *B. subtilis* induced by sub-inhibitory concentrations of drugs resulting in the expression of multidrug efflux pumps [28].

Both methods are semi-quantitative and the MIC values obtained depend on different factors, like solubility and diffusion. In liquid media the interaction of compounds with the bacterial cell should be more possible than in a solid matrix. For example, *S. aureus* growth in solid media is in a grape-type arrangement, where each cell is very close to another, impeding the contact with the compounds, while *B. subtilis* grows in long chains that can associate in palisades in solid media.

### 2.2. Diffusion Coefficient Measurements

Using the Taylor-Aris dispersion technique, the diffusion coefficient (*D*) for all compounds included in this study was determined (Table 2).

**Figure 4:**
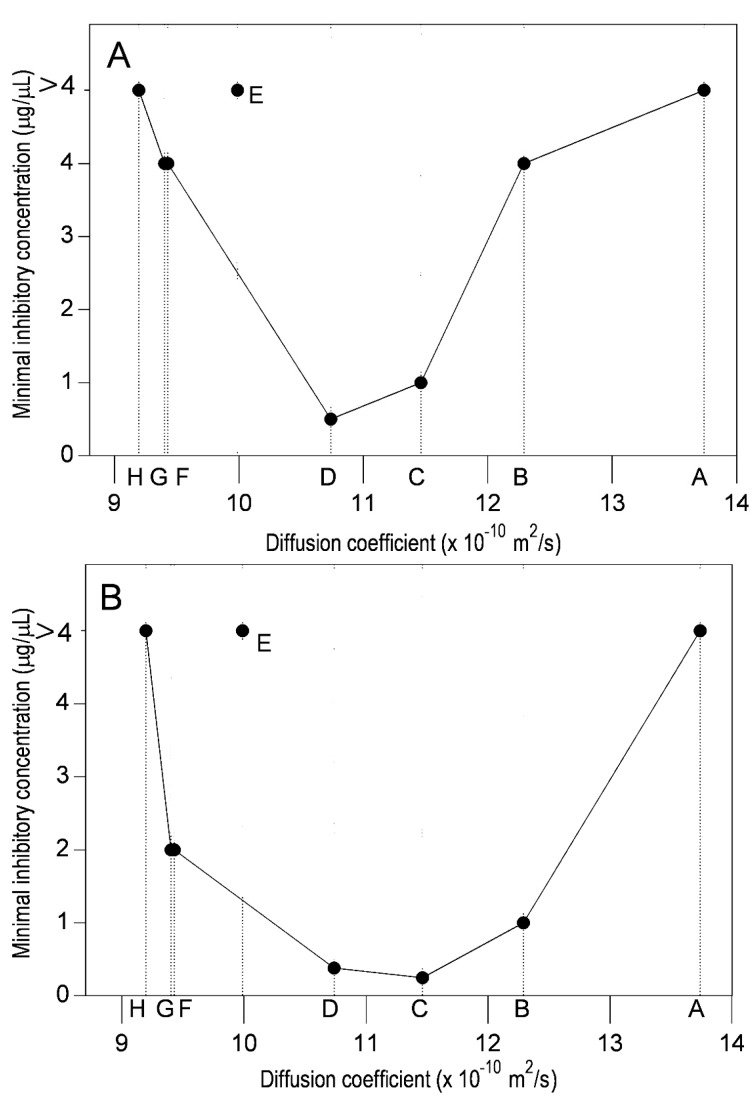
Relation between diffusion coefficient and antibacterial activity of flavonoids. (**A**) *E. coli*; and (**B**) *B. subtilis*. The compounds used were: A (3,7-*O*-dimethylgalangin), B (pinocembrin), C (galangin), D (3-*O*-methylgalangin), E (3-*O*-methylisorhamnetin), F (naringenin), G (7-*O*-methyleriodictyol), and H (quercetin), which are indicated by small uppercase letters.

Since the *D* value is related with the interaction of the solid compound with the solvent and its aggregation state in solution, compounds with a large number of hydroxyl groups will have more solvent interactions. Assuming this, the *D* value is directly related to the lipophilicity of each compound and finally to its antibacterial activity. The relation between the MIC obtained against *E. coli* and *B. subtilis*, and the D value for each flavonoid showed that the active compounds have a *D* value in the range of 9.4 × 10^−10^ to 12.3 × 10^−10^ m^2^/s, as is shown in Figure 4.

### 2.3. Lipophilicity and Antibacterial Activity

**Figure 5:**
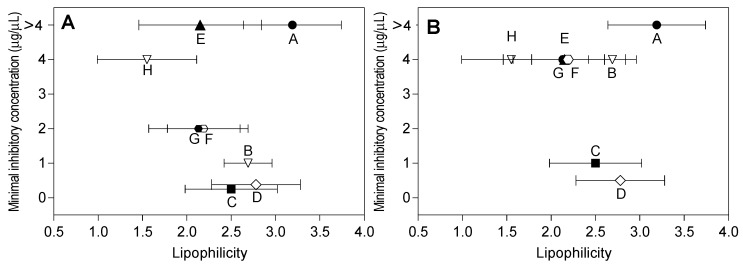
Minimal inhibitory concentration and lipophilicity relationship between flavonoids. (**A**) *B. subtilis*; and (**B**) *E. coli*. The compounds used were: A (3,7-*O*-dimethylgalangin), B (pinocembrin), C (galangin), D (3-*O*-methylgalangin), E (3-*O*-methylisorhamnetin), F (naringenin), G (7-*O*-methyleriodictyol), and H (quercetin), which are indicated by small uppercase letters.

Using the average values of the theoretical estimation of lipophilicity (Table 3), the correlations with the MIC against *B. subtilis* and *E. coli* were determined as shown in Figure 5. Considering flavanones and flavones, the log *P* values for the most active flavonoids against *B. subtilis* (Figure 5A) and *E. coli* (Figure 5B) were in the range of 2.0 to 3.3. Compound E (3-*O*-methylisorhamnetin) did not follow the observed trend with the Gram-positive bacteria. The data obtained for the D range for the active compounds (9.4 × 10^−10^ to 12.3 × 10^−10^) and the log *P* range (2.0 to 3.3), were analyzed for all compounds used in this study. Most of the active compounds had *D* and log *P* values within the predicted ranges for these parameters (Figure 5). The inactive compound 3,7-*O*-dimethylgalangin and very low antibacterial activity compounds were out of the ranges for both parameters.

## 3. Discussion

The selection of antibacterial activity determination methods is very important when the compounds to be tested are insoluble in water, as is the case for the flavonoids. In solid media, the paper disk and the cylinder methods require the diffusion of the compounds into the aqueous agar media [29]. To avoid this problem we used the direct assay method, depositing aliquots of the compound over the bacterial lawn, also allowing the rapid evaporation of methanol, the solvent used in this case. On the other hand, the antibacterial activity determination in liquid media has the problem of observed opalescence due to the insolubility of the compounds in aqueous solutions that interfere with the optical density when bacterial growth is quantified. This was solved by subtracting each optical density (O.D.) obtained with the different compound concentrations used in the assay in culture media. Despite this, at higher concentrations, it is possible that aggregations are formed between the compounds and bacteria, or forming a cumulus of dead and live bacteria as reported using galangin with *S. aureus* [30].

The antibacterial activity of the compounds galangin, naringenin, and quercetin are consistent with MIC values published using different *S. aureus* strains [25]. It is noteworthy that this is the first report of antibacterial activities against *S. aureus* of 3-*O*-methylgalangin, 7-*O*-methyleriodictyol, 3-*O*-methylisorhamnetin, and pinocembrin. In the case of 3,7-*O*-dimethylgalangin, antibacterial activity had only been informed with MIC values of 0.050 μg/μL and 0.1 μg/μL against methicillin-susceptible *S. aureus* (MSSA) and methicillin-resistant *S. aureus* (MRSA), respectively [31].

Results in solid and liquid media using the microdilution method showed that Gram-positive bacteria were slightly more susceptible to the active flavonoids than Gram-negative bacteria, as determined by the MIC. This may be due to differences in the cell wall structure between both types of bacteria [15,32]. The interaction of antibacterial compounds with the cytoplasmic membrane defines the success or failure in performing their inhibitory activity. For this reason, their physico-chemical properties, and specifically their lipophilicity, are crucial. Non-hydrophobic compounds would not cross the membrane, except through special pores, and highly-hydrophobic compounds would join too strongly to the phospholipid bilayer. Our results show that there is a close relation between lipophilicity and antibacterial activity, determined by the diffusion coefficient supporting a prediction analysis of active compounds. The most active compounds against the Gram-positive bacteria *B. subtilis* were 3-*O*-methylgalangin, galangin, pinocembrin, naringenin, and 7-*O*-methyleriodictyol, showing that the diffusion coefficient should be in the range of 9.4 × 10^−10^ to 12.3 × 10^−10^ m^2^/s, which was further supported by the lipophilicity value range. *B. subtilis* showed a wider range compared to *E. coli*. In the latter case the active compounds were only two, 3-*O*-methylgalangin and galangin. These results may be explained by the differences in the external structure of both types of bacteria, as mentioned in the introduction.

To simplify the analysis of the chemical structure and antibacterial activity, from the relationship based on their structural classification, the compounds were grouped into flavanones and flavones. Flavanones, which differ from flavones by the lack of the hetero-ring double bond, are not planar compounds. Flavones are almost planar molecules but, in contrast, flavanones are not planar, with the exocyclic phenyl ring almost perpendicular to the rest of the molecule. Despite the few compounds tested, the comparison between a flavone and flavanone with similar lipophilicity and oxygenated substitution patterns in the A and B rings (e.g., pinocembrin and 3-*O*-metilgalangin) showed that flavones have higher antibacterial activity. In addition to the planar structure, as concluded from the marked antibacterial activity of flavone, itself, several structural requirements emerged from this study.

The flavanones naringenin, 7-*O*-methyleriodictyol, and pinocembrin showed lower activity against Gram-positive bacteria compared with flavones. In Gram-positive bacteria the flavanones were more active, but when the number of hydroxyl groups in the compounds is greater, e.g., in narigenin and 7-*O*-methyleriodictyol, which have three hydroxyl groups, the compound showed less activity, while pinocembrin, with two hydroxyl groups, showed the lowest MIC. This trend was also evident with the Gram-negative bacteria. The above shows that the number of hydroxyl groups should not be an important factor, but their position proves to be a relevant factor, showing that the presence of two well-defined zones in the structure, a hydrophobic and a hydrophilic one, are necessary. Examples of this are 7-*O*-methyleriodictyol and pinocembrin, which are active against both types of bacteria and, moreover, pinocembrin is the most active, and it has hydroxyl groups in C-5 and C-7 on ring A, leaving ring B free of substituents. This suggests that the presence of two hydroxyl groups on ring A and none on ring B is a major contributing factor towards antibacterial activity. Pinocembrin also presents the highest distribution coefficient (D) and lipophilicity (log P) among the flavanones that were studied.

Among the flavones that were analyzed, the importance of the presence of hydroxyl groups for the antibacterial activity was clearly shown by comparing the activity of the series of galangin with one, two, and three hydroxyl groups in the structure, which is consistent with the structure-activity analysis recently published [33]. The most lipophilic compound, and less active as an antibacterial agent, has only one hydroxyl group (3,7-*O*-dimethylgalangin), whereas galangin was the most active, with three hydroxyl groups in its structure, followed by 3-*O*-methylgalangin, which has an intermediate lipophilicity in this series. Analyzing the remaining flavones, quercetin showed low activity, with five hydroxyl groups and high lipophilicity. As indicated before, for the compound to reach its site of action inside the bacterial cell a high hydrophilic nature would prevent it from crossing the cytoplasmic membrane. Thus, there is no lineal relation between lipophilicity and activity; instead, there is a range of lipophilicity in which the compounds can be more active, or there may be other structural factors, which could also be involved in the antibacterial activity.

The above results are consistent with the trend for flavanones where, besides the number of hydroxyl groups, their position plays an important role in the activity. This becomes apparent with the requirement of high amphipathicity of the molecule, with well-spaced hydrophobic and hydrophilic regions, which seem to be essential for the activity of the compounds. A clear example is that of galangin and its methoxylated derivative (3-*O*-methylgalangin), where it was found that the polar zone with the hydrophilic characteristics is located on ring A, while the aromatic ring B lacks substituents, constituting a lipophilic region of the molecule. It is known that the mode of antibacterial action of galangin in *S. aureus* is through the disruption of the integrity of the cytoplasmic membrane producing loss of potassium [9]. A further study showed that galangin causes aggregation of the bacterial cells of *S. aureus*, involving the cytoplasmic membrane as the target site for the activity of this compound [30].

The presence of a hydroxyl group and a methoxy group on ring B (3-*O*-methylisorhamnetin) causes a drastic decrease in activity compared to galangin; the same is true for the compound with two hydroxyls on ring B (quercetin) emphasizing the importance of these groups. For flavonoid activity in eukaryotic cells, has stressed that the interaction with the cell membrane is important [22]. Such interaction with lipids is, in most cases, limited by the polar region of the phospholipid bilayer, but the penetration depth of these compounds into the membrane depends on its structure. Most flavonoids decrease membrane fluidity. The main factor governing flavonoid-phospholipid interactions seems to be the lipophilicity of these molecules. It also appears that the presence of two hydroxyl groups located strategically on ring A and the absence of polar groups on ring B are of great importance for the antibacterial activity of flavonoids, because these groups bind strongly to the polar phospholipids region in the bacterial membrane, leaving the hydrophobic region with B ring facing the inside of the bilayer, interacting with the alkyl chains of the phospholipids. Such structural factors would explain the disruption of the membrane resulting in the bacteriolytic action of these compounds.

## 4. Materials and Methods

### 4.1. Bacterial Strains and Culture Conditions

The laboratory Gram-positive strains *Bacillus cereus* NAS 596, *Bacillus subtilis* ATCC 6633, *Bacillus coagulans* and *Staphylococcus aureus* ATCC 6538, and Gram-negative strains *Enterobacter cloacae*, *Escherichia coli* ATCC 25922, *Klebsiella pneumoniae* ATCC 13883, and *Proteus mirabilis* were used. All strains were cultivated in lysogeny broth (LB) (tryptone 10 g/L; yeast extract 5 g/L; NaCl 5 g/L) at 37 °C or in LB agar (LB broth with 1.5% solid agar or 0.75% soft agar).

### 4.2. Chemical Reagents

The natural compounds 3-*O*-methylgalangin (**7**), 3,7-*O*-dimethylgalangin (**8**), naringenin (**1**), pinocembrin (**2**), 7-*O*-methyleriodictyol (**3**) and 3-*O*-methylsorhamnetin (**6**) were isolated from the resinous exudates of *Heliotropium filifolium* (Miers) I.M. Johnst., *H. huascoense, H. sinuatum* I.M. Johnst. and *H. stenophyllum* Hook. and Arn. as described in [34,35,36,37] and *Pseudognaphalium cheiranthifolium* (Lam.) Hilliard and B.L. Burtt*,* and *Pseudognaphalium gayanum* (J. Remy) Anderb. *(*ex*-P. heterotrichium* (Phil.) Anderb. and ex*-P. robustum* (Phil.) Anderb.), as characterized in [36,38,39,40]. The purity of the isolated compounds (98%–99%) was confirmed by thin-layer chromatography (TLC) and high perfomance liquid chromatography with ultraviolet detector (HPLC-UV) analysis. The commercial flavonoid quercetin (**4**) and galangin (**5**) were also used (Sigma-Aldrich, St. Louis, MO, USA). The structures of the flavonoids used in this study are shown in Figure 1.

### 4.3. Antibacterial Activity

The antibacterial activity in solid media was determined by the agar overlay method [41]. Bacteria grown overnight in LB-broth were diluted to McFarland 0.5 (2 × 10^8^ colony forming units (cfu)/mL), and a portion of this diluate (100 μL) were mixed with molten soft agar (0.7%, 3 mL) at 55 °C. The soft agar was poured over Petri dishes containing 1.5% agar (20 mL). Two-fold dilutions of the rest samples (5 μL) in methanol were deposited over solidified agar, starting at 1 at 0.08 μg/μL (galangin, 3-*O*-methylgalangin, and 3,7-*O*-dimethylgalangin), to 4 at 0.03 μg/μL (quercetin, naringenin, 7-*O*-methylisorhamnetin, 3-*O*-methyleriodictyol, and pinocembrin). After 18 h of incubation at 37 °C, the diameter of the inhibition zone was determined. Control measurements were carried out with methanol. The maximum stock concentration used (1 and 4 mg/mL) is related to the more or less activity shown by the compounds; the exception was for 3-*O*-dimethylgalangin, whose stock concentration was the same as the most related compounds. The minimum inhibitory concentration corresponded to the minimum concentration that showed a transparent halo of growth inhibition. The MIC determination was carried out in three independent experiments.

The inhibition activity in liquid media was determined in 96-well micro-dilution plates [42], adding to each well 2 μL of an overnight bacterial culture diluted to McFarland 0.5 (2 × 10^8^ cfu/mL), 10 μL of the compound dilution, and LB-broth to complete the final volume of 150 μL. The final concentration of the different compounds ranged from 2.5 μg/μL to 0.01 μg/μL, but in the case of galangin and 3-*O*-methylgalangin, the assayed concentration range was 1 to 0.008 μg/μL. The plates were incubated stationary at 37 °C for 18 h and the density of the bacterial growth (O.D.) in each well was measured using an ELISA reader Multiskan FC at 540 nm (Thermo, Waltham, MA, USA). The determination was carried out in three independent experiments.

### 4.4. Effect of the Flavonoids on the Bacterial Growth Curve

The effect of the compounds during bacterial growth was studied following absorbance changes when different concentrations of the compounds were added to an O.D._540 nm_ 0.5 culture (mid-exponential growth). The number of viable cells was determined by plating dilutions on LB agar and after incubation for 18 h at 37 °C. 

### 4.5. Diffusion Coefficient Determination

The diffusion coefficient (*D*) was calculated using the Taylor-Aris dispersion technique [23,43]. The Taylor-Aris equipment consisted of a hollow stainless steel 10 m column (Waters Corporation, Milford, MA, USA) with an internal diameter of 0.34 mm, immersed in a thermostated water bath maintained at 25 ± 0.1 °C (Julabo, Seelbach, Germany), attached to a Merck-Hitachi L-4250 UV-VIS detector (Darmstadt, Germany), and a Merck-Hitachi L-6000 HPLC pump, equipped with a Rheodyne injector and an injection chamber of 20 μL. Calibration of the tube radius was performed with caffeine, as a primary, and urea and benzene, as secondary standards. For the determination of optimal conditions, the solvent flow was varied until the diffusion coefficient of standard caffeine did not vary with the applied pressure. Constant values for the diffusion coefficient were obtained for retention times larger than 2500 s, which corresponded to a solvent flow of 0.09 mL·min^−1^. Flavonoid solutions of concentration 5 × 10^−4^ mol·dm^−3^ were injected into the column, and the resulting Gaussian dispersion curves analyzed graphically, yielding, together with the corresponding retention times, values for the diffusion coefficients according to *D* = 0.231 *r*^2^*t*_r_/(*w*_1/2_)^2^, where *t*_r_ is the retention time, *w*_1/2_ is the mid-curve width, and the dispersion tube radius (*r*) was 3.045 × 10^−4^ m.

### 4.6. Theoretical Estimation of Lipophilicity

The log *P* values were calculated according to Moriguchi’s method (MLOGP), and the Ghose–Crippen log *P* (ALOGP) was computed by means of the Dragon Plus 5.4 software (Moriguchi octanol–water partition coefficient, Dragon 5.4, Talete, Milano, Italy). ALOGPS 2.1 software [44,45] allowed computation of ALOGPs [46], AClogP (Actelion, Allschwil, Switzerland), AB/LogP (ADME Boxes version 4.0, Pharma Algorithms, Toronto, ON, Canada), miLogP (Molinspiration Cheminformatics, Slovensky Grob, Slovakia), KOWWIN (Syracuse Research Corporation, Syracuse, NY, USA), XLOGP2, and XLOGP3 [47] through different algorithms based on structural, atomistic, topological, and electro-topological considerations.

## 5. Conclusions

Flavones and flavanones studied in this work showed good activity against Gram-positive and Gram-negative bacteria. Additionally, the results of antimicrobial activity successfully established a structure-activity relationship, in which the lipophilic characteristic was important for antibacterial activity and oxygenation patterns of flavones were important in selectivity. These findings provide further evidence that structural requirements should be considered in the series of antibacterial flavonoids and establish a good starting point in the search for new compounds in the rational development of new antibacterial agents.

## Figures and Tables

**Table 1:** Antibacterial activity of flavonoids in solid media against Gram-positive and Gram-negative bacteria.

Compound	Minimal Inhibitory Concentration in Solid Media (µg/µL) *							
	Gram-Negative Bacteria				Gram-Positive Bacteria			
	*E. cloacae*	*E. coli*	*K. pneumoniae*	*P. mirabilis*	*B. cereus*	*B. coagulans*	*B. subtilis*	*S. aureus*
**Naringenin** (4,5,7-Trihydroxyflavanone)	2.00	4.00	>4.00	2.00	2.00	2.00	2.00	>4.00
**Pinocembrin** (5,7-Dihydroxyflavanone)	1.00	4.00	1.00	4.00	2.00	1.00	1.00	>4.00
**7-*O*-Methyleriodictyol** (3′,4′,5-Trihydroxy-7-methoxyflavanone)	2.00	4.00	>4.00	0.50	2.00	1.00	2.00	4.00
**Quercetin** (3,3′,4′,5-7-Pentahydroxyflavone)	>4.00	>4.00	>4.00	0.50	2.00	2.00	>4.00	2.00
**Galangin** (3,5,7-Trihydroxyflavone)	1.00	1.00	0.50	0.25	0.25	0.25	0.25	0.50
**3-*O*-Methylisorhamnetin** (5,7,4′-Trihydroxy-3,3′-dimethoxyflavone)	>4.00	>4.00	>4.00	>4.00	2.00	1.00	>4.00	>4.00
**3-*O*-Methylgalangin** (5,7-Dihydroxy-3-methoxyflavone)	1.00	0.50	0.50	0.25	0.25	0.38	0.38	0.50
**3,7-*O*-Dimethylgalangin** (5-Hydroxy-3,7-dimethoxyflavone)	>4.00	>4.00	>4.00	>4.00	>4.00	>4.00	>4.00	>4.00

* Five microliters from each concentration was deposited on the bacterial lawn.

**Table 2:** Diffusion coefficients determined by the Taylor-Aris dispersion technique.

Compound ^a^	*t_r_*^b^ (min)		*w*_1/2_^c^ (cm)		Diffusion Coefficient ^d^ (×10^−9^)		
	1	2	1	2	1	2	Mean
**Naringenin** (4,5,7-Trihydroxyflavanone)	48.7	48.8	1.1	1.1	0.90	0.99	0.95
**Pinocembrin** (5,7-Dihydroxyflavanone)	49.5	49.2	1.0	0.9	1.10	1.35	1.23
**7-*O*-Methyleriodictyol** (3′,4′,5-Trihydroxy-7-methoxyflavanone)	50.8	51.2	1.1	1.1	0.94	0.94	0.94
**Quercetin** (3,3′,4′,5-7-Pentahydroxyflavone)	49.4	50.3	1.1	1.1	0.91	0.93	0.92
**Galangin** (3,5,7-Trihydroxyflavone)	50.7	52.1	1.0	1.0	1.13	1.16	1.15
**3-*O*-Methylisorhamnetin** (5,7,4′-Trihydroxy-3,3′-dimethoxyflavone)	49.2	49.5	1.1	1.1	0.96	1.00	0.98
**3-*O*-Methylgalangin** (5,7-Dihydroxy-3-methoxyflavone)	48.2	48.1	1.0	1.0	1.08	1.07	1.08
**3,7-*O*-Dimethylgalangin** (5-Hydroxy-3,7-dimethoxyflavone)	49.6	50.2	0.9	0.9	1.37	1.38	1.38

^a^ The flavonoid concentrations were 5 × 10^−4^ M in methanol, and 20 μL volumes were injected through a 10 m dispersion tube at 25 °C; ^b^ Retention time; ^c^ Width at half peak height detected at 268 nm; ^d^ The diffusion coefficient was calculated as *D* = 0.231*r*^2^*t_r_*/(*w_1/2_*)^2^. The mean values considered two determinations.

**Table 3:** Data of lipophilicity parameters.

Flavonoids	ALOGPs	ACLogP	AB/LogP	miLogP	ALOGP	MLOGP	KOWWIN	XLOGP2	XLOGP3	Average Lipophilicity
**Naringenin** (4,5,7-Trihydroxyflavanone)	2.47	2.50	2.33	2.12	2.30	1.45	2.61	1.57	2.39	2.19 ± 0.41
**Pinocembrin** (5,7-Dihydroxyflavanone)	2.85	2.80	2.95	2.60	2.57	2.24	3.09	2.40	2.75	2.69 ± 0.27
**7-*O*-Methyleriodictyol** (3′,4′,5-Trihydroxy-7-methoxyflavanone)	2.54	2.40	2.31	2.16	2.28	0.93	2.69	1.49	2.36	2.13 ± 0.56
**Quercetin** (3,3′,4′,5-7-Pentahydroxyflavone)	1.81	1.80	2.34	1.68	1.50	0.23	1.48	1.52	1.54	1.55 ± 0.56
**Galangin** (3,5,7-Trihydroxyflavone)	2.39	2.40	3.42	2.65	2.04	1.76	2.44	3.17	2.25	2.50 ± 0.52
**3-*O*-Methylisorhamnetin** (5,7,4′-Trihydroxy-3,3′-dimethoxyflavone)	3.00	2.45	2.55	2.27	1.82	0.73	1.96	1.72	2.82	2.15 ± 0.69
**3-*O*-Methylgalangin** (5,7-Dihydroxy-3-methoxyflavone)	3.49	2.86	3.33	2.93	2.10	2.01	2.62	3.06	2.58	2.78 ± 0.50
**3,7-*O*-Dimethylgalangin** (5-Hydroxy-3,7-dimethoxyflavone)	3.74	3.05	3.77	3.46	2.35	2.26	3.18	3.38	3.53	3.19 ± 0.55
